# mRNA vaccine effectiveness against hospitalisation due to severe acute respiratory infection (SARI) COVID-19 during Omicron variant predominance estimated from real-world surveillance data, Slovenia, February to March 2022

**DOI:** 10.2807/1560-7917.ES.2022.27.20.2200350

**Published:** 2022-05-19

**Authors:** Marta Grgič Vitek, Irena Klavs, Veronika Učakar, Marjana Vrh, Maja Mrzel, Mojca Serdt, Mario Fafangel

**Affiliations:** 1 Communicable Diseases Centre, National Institute of Public Health, Ljubljana, Slovenia

**Keywords:** vaccine effectiveness, mRNA vaccines, severe acute respiratory infection (SARI), COVID-19, Omicron variant, surveillance, Slovenia

## Abstract

For the period of predominance of SARS-CoV-2 Omicron variant in Slovenia, February to March 2022, we estimated mRNA vaccine effectiveness (VE) against severe acute respiratory infection (SARI) COVID-19 using surveillance data. In the most vulnerable age group comprising individuals aged 65 years and more, VE against SARI COVID-19 was 95% (95% CI: 95–96%) for those vaccinated with three doses, in comparison to 82% (95% CI: 79–84%) for those vaccinated with two doses. Such levels of protection were maintained for at least 6 months.

We estimated vaccine effectiveness (VE) of coronavirus disease (COVID-19) vaccines used in Slovenia against hospitalisation due to severe acute respiratory infection (SARI) COVID-19 for the period with Delta variant predominance in October 2021 [1]. Emergence of the severe acute respiratory syndrome coronavirus 2 (SARS CoV-2) Omicron variant that became predominant in Slovenia in January 2022 [2] has raised concerns about the effectiveness of COVID-19 vaccines used in the country. Our objective was to estimate the VE of the second and third dose of mRNA vaccines against SARI COVID-19 hospitalisation from February to March (weeks 5–12) 2022, by age groups and by time since the last dose.

## Case definitions and data sources

A SARI case was defined as any case of acute respiratory infection of such severity that it resulted in hospital admission [3]. A SARI COVID-19 case was defined as an individual with SARI and a positive SARS-CoV-2 PCR or rapid antigen test (RAT) result at hospital admission [3]. A case with a previous COVID-19 diagnosis was defined as an individual with a record of a positive SARS-CoV-2 PCR (or a positive RAT only, during two periods, from 21 December 2020 to 12 February 2021 and from 1 February 2022 to 27 March 2022) in the national COVID-19 database that had occurred more than 2 weeks before the week under observation. Individuals vaccinated with two or three doses were those who received the primary recommended vaccination schedule (two doses) or booster or additional dose (three doses) of mRNA vaccines used in Slovenia: Comirnaty (BNT162b2, BioNTech-Pfizer, Mainz, Germany/New York, United States) or Spikevax (mRNA-1273, Moderna, Cambridge, United States) more than 2 weeks before the week under observation. Unvaccinated individuals were defined as individuals who had not received any dose of any vaccine.

Three different sources were used to obtain the data for the analysis: weekly numbers of SARI COVID-19 cases admitted to all hospitals in Slovenia were extracted from EPISARI database [3], vaccination status of all individuals vaccinated in Slovenia was obtained from the national electronic registry of vaccinated individuals and adverse events following immunisation (eRCO) [4] and information on previous diagnosis of COVID-19 was retrieved from the national COVID-19 database. Detailed description of the three databases used is available in the Supplement to this manuscript. By using unique identifiers, we linked the data from the three databases to ascertain the vaccination status of SARI COVID-19 cases admitted to hospitals and previous diagnosis of COVID-19.

## Estimating vaccine effectiveness

To estimate VE against hospitalisation due to SARI COVID-19 for the second and third dose of mRNA vaccines by age groups and by time since vaccination, we used (i) the respective rates of SARI COVID-19 cases among individuals vaccinated with the second or third dose who had not had a previous diagnosis of COVID-19 and (ii) the respective rates of SARI COVID-19 cases among unvaccinated individuals without previous diagnosis of COVID-19.

VE was calculated using the formula: (1 − RR) × 100, where RR was the ratio of the SARI COVID-19 cases rate among those vaccinated with second/third dose, to the SARI COVID-19 cases rate among those unvaccinated.

To obtain estimates of weekly denominators (for each Monday during 8 weeks under observation) we counted individuals vaccinated with a second or third dose and without previous diagnosis of COVID-19. To obtain estimates of weekly denominators for unvaccinated individuals without previous diagnosis of COVID-19, we subtracted the number of individuals who have received at least one dose of any vaccine against COVID-19, and the number of individuals with previous COVID-19 diagnosis by each Monday for all weeks under observation, from the total number of individuals in the Central Population Registry on 1 January 2021. Then average numbers of respective weekly estimates for all these denominators were used to estimate VE for the 8-week period under observation.

## Effectiveness of the second and third dose of mRNA vaccines against SARI COVID-19

During February and March (weeks 5–12) 2022, the period of Omicron predominance [2], the overall SARI COVID-19 admissions to Slovenian hospitals among individuals aged 18 years and more was 98/100,000 population. Respective rates varied substantially by age and vaccination status. Among unvaccinated, SARI COVID-19 admissions were 343/100,000, among vaccinated with two doses of mRNA vaccines 132/100,000, and among those vaccinated with three doses 74/100,000.

For individuals without previously confirmed COVID-19, the Table shows SARI COVID-19 admission numbers and rates according to vaccination status with mRNA vaccines (two, three doses and no dose of any vaccine), age groups, and time since the last vaccine dose. Respective estimates of mRNA VE are shown.

**Table:** Effectiveness of two and three doses of mRNA vaccines against SARI COVID-19 hospitalisations among individuals without previous COVID-19 diagnosis^a^, during Omicron predominance, by age group and time since last dose, Slovenia, February–March 2022

Age group (years)	Individuals vaccinated with two doses^b^			Individuals vaccinated with three doses^b^			Unvaccinated individuals^c^			Vaccine effectiveness			
										Individuals vaccinated with two doses^b^		Individuals vaccinated with three doses^b^	
	Number of SARI COVID-19 cases^d^	Population vaccinated with two doses^b^	Rate per 100,000	Number of SARI COVID-19 cases^d^	Population vaccinated with three doses^b^	Rate per 100,000	Number of SARI COVID-19 cases^d^	Unvaccinated population	Rate per 100,000	%	95% CI	%	95% CI
Irrespective of the last dose timing													
18–49	11	124,922	8.8	5	60,627	8.3	44	154,167	28.5	69	40–84	71	27–89
50–64	42	63,231	66.4	8	83,781	9.6	163	66,685	244.4	73	62–81	96	92–98
≥ 65	275	61,019	450.7	261	225,199	115.9	641	26,031	2,462.4	82	79–84	95	95–96
Last dose ≤ 3 months ago													
18–49	2	21,429	9.3	5	55,020	9.1	44	154,167	28.5	67	0–92	68	20–87
50–64	14	8,445	165.8	3	72,696	4.1	163	66,685	244.4	32	0–61	98	95–99
≥ 65	38	9,181	413.9	133	142,585	93.3	641	26,031	2,462.4	83	77–88	96	95–97
Last dose 4–5 months ago													
18–49	4	37,322	10.7	0	4,718	0.0	44	154,167	28.5	62	0–87	100	ND
50–64	4	17,503	22.9	5	10,109	49.5	163	66,685	244.4	91	75–97	80	51–92
≥ 65	79	16,925	466.8	124	78,438	158.1	641	26,031	2,462.4	81	76–85	94	92–95
Last dose ≥ 6 months ago													
18–49	5	66,172	7.6	0	889	0.0	44	154,167	28.5	74	33–90	100	ND
50–64	24	37,283	64.4	0	976	0.0	163	66,685	244.4	74	60–83	100	ND
≥ 65	158	34,913	452.6	4	4,177	95.8	641	26,031	2,462.4	82	78–85	96	90–99

CI: confidence interval; COVID-19: coronavirus disease; mRNA: messenger ribonucleic acid; ND: not determined (95% CIs were not determined for all VE estimates of 100%); SARI: severe acute respiratory infection; SARS-CoV-2: severe acute respiratory syndrome coronavirus 2.

^a^ Individuals without previous COVID-19 diagnosis were defined as individuals without a record of a positive SARS-CoV-2 PCR (or only a positive RAT from 21 December 2020 to 12 February 2021 and from 1 February 2022 to 27 March 2022) in the national COVID-19 database more than 2 weeks before the week under observation within the SARI surveillance database known as EPISARI [3].

^b^ Individuals vaccinated with two or three doses were defined as individuals who had received two or three doses of mRNA vaccines (Comirnaty (BNT162b2 mRNA, BioNTech-Pfizer, Mainz, Germany/New York, United States) or Spikevax (mRNA-1273, Moderna, Cambridge, United States)) more than 2 weeks before the week under observation.

^c^ Unvaccinated individuals were defined as individuals who had not received any dose of any vaccine against COVID-19.

^d^ SARI COVID-19 cases were defined as all SARI cases testing positive for SARS-CoV-2 by PCR or antigen test at admission to hospitals.

Data sources: EPISARI surveillance of severe acute respiratory infections within comprehensive COVID-19 surveillance [3], national electronic registry of vaccinated individuals and adverse events following vaccinations [4], national COVID-19 dataset and Slovenian Central Population Registry.

Both older age groups (aged 50 to 64 years and 65 years and more) were better protected against hospitalisation due to SARI COVID-19, if they had already received three dose of mRNA vaccine in comparison to those who had received only two doses of mRNA vaccines. In the more vulnerable age group, aged 65 years and more, VE against SARI COVID-19 was 95% (95% CI: 95–96%) for those vaccinated with three doses in comparison to 82% (95% CI: 79–84%) for those vaccinated with two doses. For both these groups (two doses and three doses received), such level of VE was maintained for at least 6 months after the last dose.

## Discussion

The emergence of SARS CoV-2 Omicron variant resulted in the fifth wave of the COVID-19 pandemic in Slovenia and by the end of January 2022 the predominance of Omicron was almost 100% [2].

The risk of severe outcome following SARS-CoV-2 infection is substantially lower for Omicron variant vs Delta variant [5]. Recent studies reported lower effectiveness of current vaccines in preventing infections with the Omicron variant in comparison to previous variants [6-8]. However, while breakthrough infections with SARS-CoV-2 Omicron variant are quite common even after a booster dose [9], several studies reported that protection against hospitalisation was maintained, and mRNA vaccine boosters offered a high level of protection against hospitalisation and death [5,8,10-12].

Our results also showed high effectiveness of two doses of mRNA vaccines and very high effectiveness of three doses against SARI COVID-19 hospitalisations across all age groups studied. To receive the third dose of mRNA vaccine is especially important for elderly people, aged 65 years or more, who are at the highest risk for serious disease that may result in hospitalisation. In this group, VE against SARI COVID-19 was 82% (95% CI: 79–84%), if vaccinated with two doses of mRNA vaccine, and 95% (95% CI: 95–96%) with three doses. It should be noted that for both groups, those who received two and those who received three doses, such high VE lasted for at least 6 months after the last dose received. This was unlike the SARI COVID-19 waning immunity we observed during the period of Delta predominance, in a previous VE study using the same methodology and data sources [1]. Understanding how much longer the high protection against Omicron-related SARI COVID-19 hospitalisation will last, will only be possible in the future, when the number of people vaccinated with the third dose more than 6 months ago will accumulate. Similar results with respect to protection against hospitalisation due to Omicron-related COVID-19 for elderly individuals (aged 70 years and older) were reported from Finland (VE of 91% and 76%, 14–90 and 91–180 days after second dose, and 95%, 14–60 days after third dose) [11].

In contrast to our results, waning of protection against Omicron-related COVID-19 hospitalisation with time since vaccination with either two or three doses was reported by some [7,11,12], while strong and durable protection after the second mRNA dose and even more robust protection after a booster dose was also reported by a recent study from Qatar [13].

While quantified validations of the three databases are not available, they provide real-world national surveillance data collected from all Slovenian hospitals (EPISARI), and data from official national registries (eRCO and COVID-19 databases) [3,4]. The three databases are described in detail in the Supplement to this manuscript. It was important to exclude prior COVID-19 confirmed cases. The analysis including prior COVID-19 confirmed cases underestimated VE (data not shown). We are aware that we could not exclude all prior COVID-19 cases, as some were not diagnosed. Asymptomatic individuals and those with mild symptoms might not have sought testing and all individuals with a positive self-test result for SARS-CoV-2 infection may have not asked for confirmatory testing although it was strongly recommended.

Surveillance of SARI COVID-19 cases within EPISARI has some limitations, which have been described in detail previously [1,3]. However, it is rather unlikely that SARI COVID-19 cases admissions to hospitals, the outcome used in the analysis, would not be ascertained correctly in most cases and reported. Also, data on vaccination against COVID-19 in eRCO might not be 100% accurate. However, many vaccination providers’ data entry errors were identified and corrected swiftly, because the vaccination status recorded in eRCO was used for issuing digital COVID-19 certificates, which were essential for people during periods of restricting non-pharmaceutical interventions. Thus, we believe that vaccination against COVID-19 data used in our analyses were of good quality and well representative for the population of Slovenia aged 18 years or more. Good completeness of vaccination data was also stimulated by reimbursing only the vaccinations reported to eRCO. Also, testing behaviour and preventive behaviour (e.g. adherence to non-pharmaceutical measures) may have differed between vaccinated and unvaccinated, which could result in, to some extent, biased VE estimates. Finally, as no information on comorbidities of SARI COVID-19 cases was available, it was difficult to assess to what extent the estimated reduced VE in younger age groups occurred because of low numbers of individuals at high risk of severe disease. Thus, those VE estimates by age group and time since the last dose that are based on very small numbers of SARI COVID-19 cases should be interpreted with caution. Additional studies focusing on high-risk individuals among the younger population are warranted.

## Conclusion

Our results show that during the period of Omicron predominance, vaccination with two doses of mRNA vaccines offers good protection against hospitalisation due to SARI COVID-19, which is significantly enhanced by a third dose in both older (aged 50 to 64 years and 65 years and more) age groups. The third dose is especially important for protection of the most vulnerable group, individuals aged 65 years or more. It is relevant for national vaccination policy planning that in this age group such solid protection seems not to decline for at least 6 months after the last dose.

## Supplementary Data

401000 Supplement
