# Supplementary material for: mRNA vaccine effectiveness against hospitalisation due to severe acute respiratory infection (SARI) COVID-19 during Omicron variant predominance estimated from real-world surveillance data, Slovenia, February to March 2022
Source: Euro Surveill. 2022 May 19;27(20):2200350. doi: 10.2807/1560-7917.ES.2022.27.20.2200350 (PMC9121658; doi:10.2807/1560-7917.ES.2022.27.20.2200350)
Supplement: Supplement [file 2200350_Supplement.pdf]

This supplementary material is hosted by Eurosurveillance as supporting information alongside the article “**mRNA vaccine effectiveness against hospitalisation due to severe acute respiratory infection (SARI) COVID-19 during Omicron variant predominance estimated from real-world surveillance data, Slovenia, February to March 2022**”
on behalf of the authors, who remain responsible for the accuracy and appropriateness of the content. The same standards for ethics, copyright, attributions and permissions as for the article apply. Supplements are not edited by Eurosurveillance and the journal is not responsible for the maintenance of any links or email addresses provided therein.

SUPPLEMENTARY INFORMATION ON DATA SOURCES USED

1. Slovenian national EPISARI database

To inform national COVID-19 prevention and control measures, in March 2020, the National Institute of Public Health (in Slo.: Nacionalni inštitut za javno zdravje - NIJZ) started developing syndromic epidemiological surveillance of severe acute respiratory infections (SARI), named EPISARI, within comprehensive surveillance of COVID-19 in collaboration with all 15 Slovenian acute care hospitals. The EPISARI Network was established with EPISARI contact points in participating hospitals. The main objective was to monitor severe COVID-19 morbidity by monitoring weekly numbers of SARI confirmed COVID-19 cases admissions to hospitals and intensive care units (ICUs). Additional objectives were to monitor trends in weekly numbers of: (a) all SARI admissions, (b) SARI admissions tested for SARS-CoV-2 infection, (c) COVID-19 deaths in hospitals, (d) admissions of patients with confirmed SARS CoV-2 infection for other reasons (e. g. trauma, delivery, etc. without SARI at admission), and (e) diagnoses of COVID-19 during hospitalisation, either community-acquired or healthcare-associated. In February 2021, participation in EPISARI became mandatory for all hospitals according to the Law on additional interventions for diminishing COVID-19 impact (1). All 29 Slovenian hospitals have been participating in the EPISARI Network since the beginning of April 2021.

SARI was defined as any acute respiratory infection of such severity that it results in admission to hospital. SARI confirmed COVID-19 case admission was defined as a SARI case admitted to hospital with a positive SARS-CoV-2 polymerase chain reaction (PCR) or positive antigen test result at admission (2). Confirmed COVID-19 case admission for other reasons, not SARI, was defined as admission of a patient with a positive SARS-CoV-2 PCR or positive antigen test result at admission when the patient did not have symptoms and signs of SARI at admission. The discrimination between community-acquired and healthcare-associated COVID-19 cases diagnosed during hospitalisation is at the discretion of the EPISARI contact points in hospitals, who had been made aware of respective European Centre for Disease Prevention and Control (ECDC) surveillance definitions for COVID-19 (3).

The data collection procedures in hospitals are at their discretion. Hospital EPISARI contact points report weekly EPISARI data to NIJZ, by each Wednesday for the preceding week (defined from 00:00 on Monday morning to 24:00 on Sunday evening). The data include weekly numbers of: SARI cases admitted to hospital and to ICUs; SARI cases admitted to hospital and to ICUs tested for SARS-CoV-2 infection; SARI confirmed COVID-19 cases admitted to hospital and to ICUs; confirmed COVID-19 cases admitted to hospital for other reasons, not SARI; diagnoses of community-acquired COVID-19 cases and diagnoses of healthcare-associated COVID-19 cases during hospitalisation. In addition, a unique national identifier for

each COVID-19 case containing information on age and sex of the patient is reported according to the Law on healthcare datasets (4).

The magnitude of severe COVID-19 morbidity is the key factor for informed and proportional public health response. Within the comprehensive Slovenian COVID-19 surveillance system, enhanced national surveillance of SARI (EPISARI) is a “surveillance tool” to above all monitoring trends in weekly absolute numbers or rates of SARI confirmed COVID-19 admissions to all Slovenian hospitals. This is essential in the context of evolving epidemic waves, changing vaccination coverage, changes in predominant SARS-CoV-2 variants circulating and threatening emergence of new SARS-CoV-2 variants of concern that might overcome previous SARS-CoV-2 infection or vaccine-induced immunity against severe COVID-19 disease.

SARI surveillance was recommended as one of the approaches within comprehensive COVID-19 surveillance by ECDC (5). Similarly, the World Health Organisation (WHO) provided guidance for adapting influenza SARI sentinel surveillance systems to complement COVID-19 surveillance (6).

The strength of EPISARI is the national coverage. Limitations of EPISARI may include variation in methods for SARI and different COVID-19 cases ascertainment between hospitals as the data collection process is at the discretion of individual hospitals, and possible validity issues of the data submitted to the NIJZ. Some SARI cases as well as some COVID-19 cases may be misclassified, especially community-acquired and healthcare-associated COVID-19 cases. Especially SARI cases and community-acquired and healthcare-associated COVID-19 cases may also be underreported from some hospitals. However, it is rather unlikely that SARI COVID-19 cases admissions to hospitals, the outcome used in the analysis for the above-mentioned paper would not be ascertained correctly and reported.

To conclude, we believe that EPISARI COVID-19 admissions to hospitals data are of good enough quality and well representative of the Slovenian population old 18 years or more, although no formal validation was performed.

We have been publishing weekly EPISARI reports on the NIJZ website (https://www.nijz.si/sl/dnevno-spremljanje-okuzb-s-sars-cov-2-covid-19). Also, a brief description of our national EPISARI surveillance system with selected EPISARI results for the period of 13 to 37 week 2021 were published in Eurosurveillance (7).

2. National Electronic registry of vaccinated individuals and adverse events following immunisation (eRCO)

In 2017, according to the Healthcare Databases Act (4), NIJZ launched the Slovenian national Electronic registry of vaccinated individuals and adverse events following immunization (AEFI) (eRCO) within eHealth (8). The objectives were to monitor the implementation of vaccination according to the Communicable Diseases Act (9) and annual national immunization programmes (10) and to monitor vaccination coverage and AEFI.

According to the Rules on certificates, records and reports on vaccination and vaccination adverse effects and errors (11), vaccination providers (public and private) are obliged to keep records of completed vaccinations and protection with specific immunoglobulins as well as AEFI and report to eRCO. The administrators of software solutions at vaccination providers adjusted their software programs so that

standardised recording of vaccination and AEFI data as well as data transfer to the eRCO central database is ensured. To minimize error, education on reporting protocols is routinely performed.

eRCO database includes the following information: unique national identifier, number of health insurance, name and surname of vaccinated person, residence data, date of vaccination, vaccine used, method and location of application, date of expiration, serial number of vaccine used, dose number, and the name of the health care provider who performed the vaccination.

As part of the national vaccination campaign against COVID-19 in Slovenia, the reporting of all providers of vaccination against COVID-19 to eRCO was established under the intense supervision of the Health Inspectorate. This provided for accurate real-time monitoring of vaccination against COVID-19 coverage and regular publishing of the results through an interactive dashboard on the NIJZ website (12). It also provided for the issuance of digital COVID-19 certificates (DCC) to vaccinated persons.

Due to the enhanced scrutiny of vaccination data by all stakeholders and their linkage to digital COVID-19 certificates essential for day-to-day activities of individuals during periods of non-pharmaceutical interventions nationwide, vaccination providers identified and corrected data-entry errors swiftly. Possible limitations regarding the data quality may include some remaining data-entry errors. In addition to legal obligation to report, the good completeness of data on vaccination against COVID-19 was additionally stimulated by reimbursing only the vaccinations reported to eRCO.

To conclude, we believe that our data on vaccination against COVID-19 are of good enough quality and well representative of the Slovenian population old 18 years or more, although no formal validation was performed.

3. National COVID-19 database

National COVID-19 database is a subset of National database on communicable diseases (Evidenca nalezljivih bolezni) based on the Communicable Diseases Act (9) and defined by the Healthcare Databases Act (4).

in March 2020, with the epidemic of COVID-19 declared in Slovenia, mandatory reporting of every confirmed case of SARS-CoV-2 infection in accordance with the case definition (2) was introduced. The objective was to monitor trends in COVID-19 cases. The COVID-19 surveillance results are published daily on the NIJZ website (13).

A confirmed COVID-19 case was defined as an individual in whom the infection with SARS-CoV-2 was confirmed by PCR. During the periods from 21st December 2020 (52nd week 2020) to 12th February 2021 (6th week 2021) and from 1st February 2022 (5th week 2022) onwards, a confirmed COVID-19 case was also defined as an individual in whom the infection with SARS-CoV-2 was confirmed only by a rapid antigen test (RAT).

NIJZ developed a system for the digitalised reporting of confirmed cases by all microbiological laboratories performing PCR testing of clinical samples for SARS-CoV-2 infection in Slovenia to the Central Patient Data Registry (CPDR) at NIJZ. In December 2020, this system was upgraded to allow for digital reporting of positive SARS-CoV-2 RAT results from all providers of RAT testing. Information on all SARS-CoV-2 infections cases are extracted daily from the CPDR and incorporated into the national COVID-19 database.

In addition to legal obligation to report, the completeness of COVID-19 cases in the national COVID-19 database was stimulated by the reimbursement of testing costs only, if testing data was reported to the CPDR. Since the data about positive SARS-CoV-2 testing results entered in the CPDR were also the basis for issuing of digital COVID-19 certificates (DCC) for individuals with previous COVID-19, data entry mistakes by reporting testing providers were identified and corrected swiftly.

It is a fact that not all previous COVID-19 cases are recorded in the COVID-19 database, as not all SARS-CoV-2 infections were diagnosed. Some infected individuals were asymptomatic or had only mild symptoms and were not tested for SARS-CoV-2. In addition, all individuals with positive self-test result for SARS-CoV-2 infection may have not asked for confirmatory testing. However, we believe that a great majority of previous COVID-19 cases were diagnosed and reported to the CPDR and consequently recorded in the national COVID-19 database.

To conclude, we believe that our COVID-19 data are of good enough quality and well representative of the Slovenian population old 18 years or more, although no formal validation was performed.

References

1. Additional interventions for diminishing COVID-19 impact Act [Zakon o dodatnih ukrepih za omilitev posledic COVID-19]. Uradni list RS št. 15/2021. [Accessed: 9 Sep 2021]. Available at: https://www.uradni-list.si/glasilo-uradni-list-rs/vsebina?urlurid=2021315
2. European Centre for Disease Prevention and Control (ECDC). Case definition for coronavirus disease 2019 (COVID-19), as of 3 December 2020. Stockholm: ECDC; 2020. [Accessed: 27 Aug 2021]. Available at: https://www.ecdc.europa.eu/en/covid-19/surveillance/case-definition
3. European Centre for Disease Prevention and Control (ECDC). Surveillance definitions for COVID-19 Stockholm: ECDC; 2020. [Accessed: 27 Aug 2021]. Available at: https://www.ecdc.europa.eu/en/covid-19/surveillance/surveillance-definitions
4. Healthcare Databases Act [Zakon o zbirkah podatkov s področja zdravstvenega varstva]. Uradni list RS št. 65/2000. [Accessed: 11 May 2022]. Available at: http://pisrs.si/Pis.web/pregledPredpisa?id=ZAKO1419
5. European Centre for Disease Prevention and Control (ECDC). Strategies for the surveillance of COVID-19. Stockholm: ECDC; 2020. [Accessed: 27 Aug 2021]. Available at: https://www.ecdc.europa.eu/sites/default/files/documents/COVID-19-surveillance-strategy-9-Apr-2020.pdf
6. World Health Organization (WHO). Maintaining surveillance of influenza and monitoring SARS-CoV-2 – adapting Global Influenza surveillance and Response System (GISRS) and sentinel systems during the COVID-19 pandemic: Interim guidance. Geneva: WHO; 2020 (WHO/2019-nCoV/ Adapting_GISRS/2020.1). [Accessed: 27 Aug 2021]. Available at: https://www.who.int/publications/i/item/maintaining-surveillance-of-influenza-and-monitoring-sars-cov-2-adapting-global-influenza-surveillance-and-response-system-(gisrs)-and-sentinel-systems-during-the-covid-19-pandemic
7. Klavs I, Serdt M, Učakar V, Grgič-Vitek M, Fafangel M, Mrzel M, Berlot L, Glavan U, Vrh M, Kustec T, Simoniti M, Kotnik-Kevorkijan B, Lejko-Zupanc T, the EPISARI Network. Enhanced national surveillance of severe acute respiratory infections (SARI) within COVID-19 surveillance, Slovenia,

weeks 13-37 2021. Euro Surveill. 2021;26(42):pii=2100937. Available at: https://www.eurosurveillance.org/content/10.2807/1560-7917.ES.2021.26.42.2100937
8. National Institute of Public Health (NIJZ). Electronic Registry of vaccinated individuals and adverse events following vaccinations [Elektronski register cepljenih oseb in neželenih učinkov po cepljenju]. [Accessed: 2 Dec 2021]. Available at: https://www.nijz.si/sl/elektronski-register-cepljenih-oseb-in-nezelenih-ucinkov-po-cepljenju-erco
9. Communicable Diseases Act [Zakon o nalezljivih boleznih]. Uradni list RS, št. 33/06. [Accessed: 11 May 2022]. Available at: http://www.pisrs.si/Pis.web/pregledPredpisa?id=ZAKO433
10. National immunization programme [Program cepljenja in zaščite z zdravili za leto 2021]. Available at: https://www.nijz.si/sl/program-cepljenja-in-zascite-z-zdravili-2021
11. Rules on certificates, records and reports on vaccination and vaccination adverse effects and errors [Pravilnik o potrdilih, vodenju evidenc in zagotavljanju podatkov o cepljenju, neželenih učinkih po cepljenju in zdravstvenih napakah pri cepljenju]. Uradni list RS, št. 24/2017. [Accessed: 11 May 2022]. Available at: http://www.pisrs.si/Pis.web/pregledPredpisa?id=PRAV12986
12. NIJZ. Vaccination against COVID-19 in Slovenia [Cepljenje proti covid-19 v Sloveniji]. Available at: https://app.powerbi.com/view?r=eyJrIjoiYWQ3NGE1NTMtZWJkMi00NzZmLWFiNDItZDc5YjU5MGRkOGMyIiwidCI6ImFkMjQ1ZGFlLTQ0YTAtNGQ5NC04OTY3LTVjNjk5MGFmYTQ2MyIsImMiOjl9
13. NIJZ. Daily surveillance of SARS-CoV-2 infections [Dnevno spremljanje okužb s SARS-CoV-2 (COVID-19)]. Available at https://www.nijz.si/sl/dnevno-spremljanje-okuzb-s-sars-cov-2-covid-19
